# Pharmacological characterisation of anti-inflammatory compounds in acute and chronic mouse models of cigarette smoke-induced inflammation

**DOI:** 10.1186/1465-9921-11-126

**Published:** 2010-09-18

**Authors:** Wing-Yan Heidi Wan, Abigail Morris, Gillian Kinnear, William Pearce, Joanie Mok, Daniel Wyss, Christopher S Stevenson

**Affiliations:** 1 Respiratory Disease Area, Novartis Institutes for BioMedical Research, Wimblehurst Road, Horsham, RH12 5AB, UK; 2 Respiratory Pharmacology Group, Pharmacology and Toxicology Section, National Heart and Lung Institute, Centre for Integrative Mammalian Physiology and Pharmacology, Centre of Respiratory Infection, Imperial College School of Medicine, Sir Alexander Fleming Building, London SW7 2AZ, UK; 3 Current Address: Hoffmann-La Roche Inc., Inflammation Discovery, 340 Kingsland Street, Nutley, NJ, USA

## Abstract

**Background:**

Candidate compounds being developed to treat chronic obstructive pulmonary disease are typically assessed using either acute or chronic mouse smoking models; however, in both systems compounds have almost always been administered prophylactically. Our aim was to determine whether the prophylactic effects of reference anti-inflammatory compounds in acute mouse smoking models reflected their therapeutic effects in (more clinically relevant) chronic systems.

**Methods:**

To do this, we started by examining the type of inflammatory cell infiltrate which occurred after acute (3 days) or chronic (12 weeks) cigarette smoke exposure (CSE) using female, C57BL/6 mice (n = 7-10). To compare the effects of anti-inflammatory compounds in these models, mice were exposed to either 3 days of CSE concomitant with compound dosing or 14 weeks of CSE with dosing beginning after week 12. Budesonide (1 mg kg^-1^; i.n., q.d.), roflumilast (3 mg kg^-1^; p.o., q.d.) and fluvastatin (2 mg kg^-1^; p.o., b.i.d.) were dosed 1 h before (and 5 h after for fluvastatin) CSE. These dose levels were selected because they have previously been shown to be efficacious in mouse models of lung inflammation. Bronchoalveolar lavage fluid (BALF) leukocyte number was the primary endpoint in both models as this is also a primary endpoint in early clinical studies.

**Results:**

To start, we confirmed that the inflammatory phenotypes were different after acute (3 days) versus chronic (12 weeks) CSE. The inflammation in the acute systems was predominantly neutrophilic, while in the more chronic CSE systems BALF neutrophils (PMNs), macrophage and lymphocyte numbers were all increased (p < 0.05). In the acute model, both roflumilast and fluvastatin reduced BALF PMNs (p < 0.01) after 3 days of CSE, while budesonide had no effect on BALF PMNs. In the chronic model, therapeutically administered fluvastatin reduced the numbers of PMNs and macrophages in the BALF (p ≤ 0.05), while budesonide had no effect on PMN or macrophage numbers, but did reduce BALF lymphocytes (p < 0.01). Roflumilast's inhibitory effects on inflammatory cell infiltrate were not statistically significant.

**Conclusions:**

These results demonstrate that the acute, prophylactic systems can be used to identify compounds with therapeutic potential, but may not predict a compound's efficacy in chronic smoke exposure models.

## Background

Chronic obstructive pulmonary disease (COPD) is a leading cause of hospitalizations and death worldwide. The most common cause of COPD is chronic smoking, which elicits a repetitive inflammatory insult that is thought to lead to airway remodeling and, consequently, to the accelerated lung function decline that characterizes the disease. Unlike other chronic inflammatory airway diseases like asthma, there are currently no therapeutic approaches (e.g., glucocorticoids) that can attenuate the inflammation associated with COPD. This suggests that there is something different about the molecular mechanisms regulating the cigarette smoke-induced inflammation associated with the disease, which at present is not understood.

Preclinical *in vivo *models of cigarette smoke-induced lung inflammation are commonly used to investigate prospective disease mechanisms and evaluate the efficacy of candidate compounds. Exposure of laboratory animals to cigarette smoke can recapitulate many of the central features of COPD, including a slowly resolving and steroid-resistant inflammation, mucus production, airway remodeling, emphysema and changes in lung function [1-4]. Although these models use the primary etiological factor to mimic several COPD-like changes, it is difficult to determine how reliable these models are for predicting the therapeutic efficacy of candidate compounds. For instance, while steroids lack efficacy in both the preclinical models and the clinic, approaches aimed at neutralizing TNF-alpha work in the preclinical models, but do not work in the clinic. In the latter example, a possible reason for the lack of translation is that in the preclinical models genetically modified mice deficient for the TNF-alpha receptors were used and thus, in these animals the initiation of the inflammatory response to cigarette smoke exposure (CSE) was attenuated [5,6]. This was clearly a different situation to that in the clinic where an anti-TNF-alpha antibody lacked the ability to affect the progression of ongoing disease [7].

In most studies, compounds which have efficacy in acute systems also have efficacy in chronic models, too. The caveat to this is that most preclinical investigations have focused on characterizing the effects of candidate mechanisms under prophylactic conditions (using either GM mice or compounds) whether in acute or chronic CSE models [2,8-13]. Unfortunately, this approach does not closely resemble the clinical scenario where patients are treated after chronic lung inflammation has already developed. Additionally, the inflammatory response to CSE appears to be bi-phasic, with an initial neutrophilic infiltrate peaking within one week of exposures. This is subsequently followed by a more pronounced inflammation after one month of CSEs with progressive increases in neutrophils, macrophages and lymphocytes migrating to the airways [1,14]. The different kinetics and types of infiltrate suggests that there are potentially different mechanisms driving the two phases of this response; thus, a compound's efficacy may be different in an acute, prophylactic (< one week) versus chronic, therapeutic (> one month) model. This concept is supported by the observation that TLR4 knockout mice are partially protected from developing lung inflammation after acute CSE, but were not protected after chronic CSEs [15].

As such, the aim of this study was to compare the prophylactic and therapeutic effects of three broad spectrum anti-inflammatory compounds in acute and chronic CSE models, respectively. We focused on three compounds with distinct mechanisms of action - a glucocorticoid (budesonide), a phosphodiesterase (PDE) 4 inhibitor (roflumilast) and a statin (fluvastatin). As one of the primary functions of preclinical disease models is to assess the potential efficacy of candidate compounds, ideally one would examine the same endpoints in the models as in the clinic. Typically, early proof-of-concept studies for COPD anti-inflammatory strategies in man assess inflammatory cell numbers in biofluids such as bronchoalveolar lavage fluid (BALF) or induced sputum, while longer term clinical studies examine changes in lung functioning. As the latter changes are difficult to model in small animals, we focused on assessing the effects of these anti-inflammatory compounds on CSE-induced changes in BALF inflammatory cell numbers.

## Methods

### Materials

C57BL/6 mice were obtained from Charles River UK. Budesonide [16,17-Butylidenebis(oxy)-11,21-dihydroxypregna-1,4-diene-3,20-dione] was purchased from Sigma. Roflumilast [3-(cyclopropylmethoxy)-N-(3, 5-dichloropyridin-4-yl)-4-(difluoromethoxy) benzami] and fluvastatin [(3R, 5S, 6E)-7-[3-(4-fluorophenyl)-1-(propan-2-yl)-1H-indol-2-yl]-3, 5-dihydroxyhept-6-enoic acid] were made in-house (Novartis Institutes for BioMedical Research, Basel, Switzerland). University of Kentucky Research Cigarettes (brand 1R3F) were obtained from the University of Kentucky (Louisville, KY, USA).

### Animal Maintenance Conditions

Female, C57BL/6 mice (16-20 g) were housed in rooms maintained at constant temperature (21 ± 2°C) and humidity (55 ± 15%) with a 12 h light cycle and 15 - 20 air changes per h. Ten animals were housed per cage containing two nest packs filled with grade 6 sawdust (Datesand, Manchester, UK), nesting material (Enviro-Dri, Lillico, UK), maxi fun tunnels and Aspen chew blocks (Lillico, UK) to provide environmental enrichment. Animals were allowed food, RM1 Pellets, (SDS UK Ltd.) and water ad libitum.

### Statement on Animal Welfare

Studies described herein were performed under a Project License issued by the United Kingdom Home Office and protocols were approved by the Local Ethical Review Process at Novartis Institutes for BioMedical Research, Horsham.

### Cigarette smoke exposure methodology

Cigarette smoke and sham exposures were performed as previously described [10]. Mice were exposed to 4 cigarettes per exposure period, which we had previously shown to elicit a submaximal inflammatory response [10]. Sham, age- and sex-matched control animals were exposed to room-air pumped into the exposure chambers for the same duration of time (approximately 45 minutes per exposure period).

### Comparing inflammatory cell infiltrate after acute or chronic CSE

Mice were exposed as described above once a day for either 3 days or 5 days per week for 12 weeks. Animals were sacrificed with an overdose of terminal anesthetic (sodium pentobarbitone 200 mg i.p.) followed by exsanguination 24 hours after the last exposure. There were sham, time-matched controls for each time point.

### Assessing compound efficacy in models of acute CSE-induced inflammation

For the acute CSE model, the CSE regimen was performed as described above once a day and for 3 consecutive days. For studies with budesonide, the mice were dosed with either budesonide (1 mg kg^-1^) or vehicle (saline with 2% NMP) 1 hour before each air or smoke exposure by intranasal (i.n.) administration under short-acting anaesthetic as described previously [10]. For studies with roflumilast and fluvastatin, the mice were dosed with either roflumilast (3 mg kg^-1^) or fluvastatin (2 mg kg^-1^) or vehicle (0.5% CMC) *per os *(p.o.) 1 hour before and (for fluvastatin-treated and vehicle control mice) 5 hours after each air or smoke exposure. The doses and dosing schedule for each compound were based on those that we and others have previously shown to be effective in other preclinical mouse models [9,13,16,17]. Twenty-four hours after the last exposure, animals were sacrificed with an overdose of terminal anesthetic (sodium pentobarbitone 200 mg i.p.) followed by exsanguination.

### Assessing compound efficacy in models of chronic CSE-induced inflammation

For the chronic CSE model, the CSE regimen was performed as described above once a day, 5 days a week, for 14 weeks. During weeks thirteen and fourteen, mice were dosed with compounds or vehicles (as described above) concurrent with CSE. As before, animals were sacrificed with an overdose of terminal anaesthetic (sodium pentobarbitone 20 mg i.p.) followed by exsanguination 24 hours after the last exposure.

### Preparation of bronchoalveolar lavage fluid (BALF)

After animals were sacrificed, BALF was collected, processed, and BALF inflammatory cell numbers determined as described previously [10].

### Statistical Analysis

All data are presented as Mean ± Standard Error of Mean (SEM). For time course studies, a Student's t-test was used comparing all smoke-exposed animals to their corresponding time-matched sham-exposed controls. For the compound studies, a one-way ANOVA with Dunnett correction for multiple comparisons was used. A P value of less than 0.05 was considered significant. Power calculations were based on t-tests, assuming unequal variances (Satterthwaite approximation), and were based on group means and standard deviations derived from historical data. All sample sizes were based on 80% power with a two-sided alpha = 0.05. Calculations were performed using the software package NQUERY ADVISOR.

## Results

### Time-dependent changes in BALF inflammatory cell numbers over 3 months of CSE

**Figure 1:**
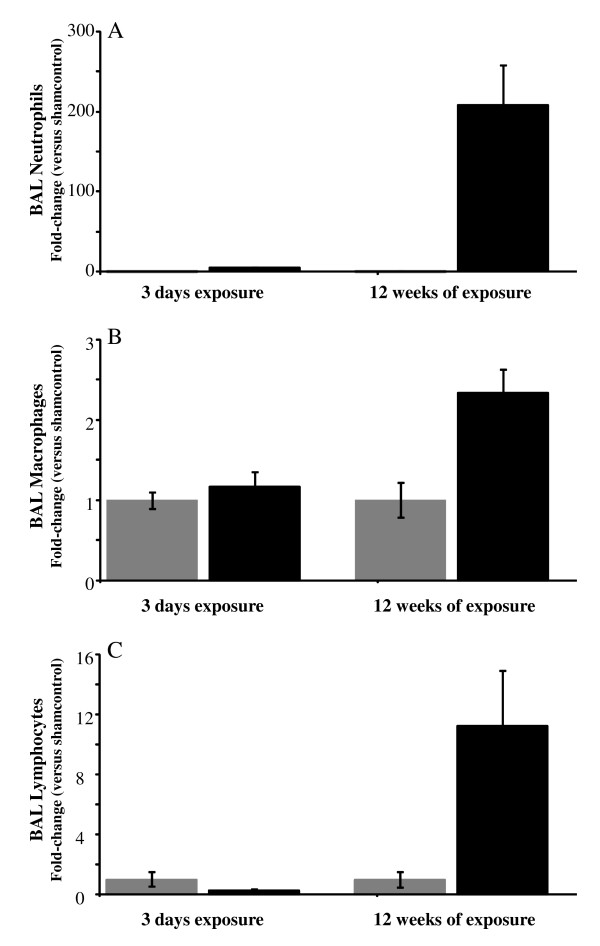
**Comparison of inflammatory cell profile after acute versus chronic CSE**. Acute (3 days) and chronic (12 weeks) CSE increased BALF neutrophils (A); however, only chronic CSE increased the numbers of BALF macrophages (B), and lymphocytes (C) in C57BL/6 mice. Data is presented as the fold-increase in the numbers of cells recovered in the BALF compared to the average of each respective sham-exposed control group. Data from smoke-exposed mice are represented by black bars and data from sham controls represented by gray bars. Data plotted as the mean ± sem with an n = 8-10 for each group. Significance (* = p < 0.05, ** = p < 0.01, *** = p < 0.001) was determined versus sham control group.

In a previous study we confirmed the bi-phasic nature of the inflammatory response to CSE over a 26 week period (data not shown). The data in figure 1, was from a separate study comparing the inflammatory phenotypes that are observed after an acute (3 days) or chronic (12 weeks) exposure period. Both acute and chronic CSE increased the numbers of BALF neutrophils recovered (Figure 1A), although it's clear chronic exposure led to a greater increase relative to each groups' respective sham controls. The numbers of neutrophils increased more than 5-fold over the 2.2 ± 0.4 × 10^3 ^cells mL^-1 ^recovered in the sham-exposed controls (p > 0.01) after 3 days of CSE; however, there was more than a 200-fold increase over the 1.7 ± 0.9 × 10^2 ^cells mL^-1 ^recovered in the sham-exposed mice after 12 weeks of CSE (p > 0.001). Increases in BALF macrophages (Figures 1B), and lymphocytes (Figures 1C) were only observed after chronic CSE. After 3 days of CSE, there were no significant increases over the numbers of macrophages (9.7 ± 1.0 × 10^4 ^cells mL^-1^) or lymphocytes (1.6 ± 0.8 × 10^3 ^cells mL^-1^) recovered in the BALF of sham-exposed mice. After 12 weeks of CSE, however, the numbers of macrophages increased more than 2-fold over the 4.2 ± 0.9 × 10^4 ^cells mL^-1 ^recovered in the sham-exposed mice (p > 0.01). Similarly, BALF lymphocyte numbers increased more than 10-fold over the 3.0 ± 1.1 × 10^3 ^cells mL^-1 ^recovered in the sham-exposed mice (p > 0.01).

### Effect of prophylactically administered anti-inflammatory compounds on CSE-induced acute inflammation

**Figure 2:**
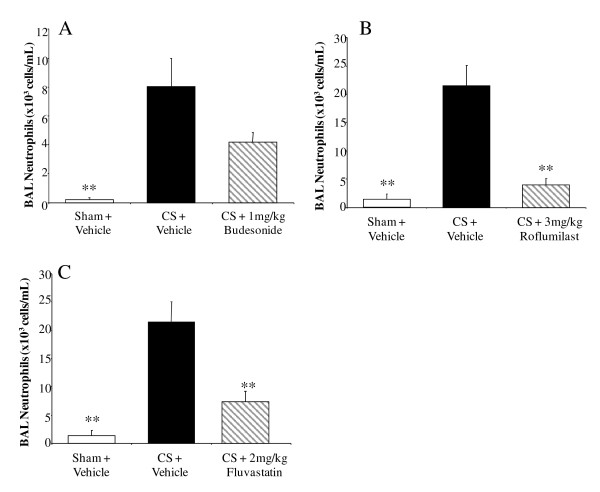
**The effect of budesonide, roflumilast and fluvastatin on acute CSE-induced neutrophil infiltrate**. (A) Budesonide (i.n., q.d.) had no effect on CSE-induced neutrophil infiltrate in mice after 3 days of exposure. (B) Roflumilast (p.o., q.d.) and (C) Fluvastatin (p.o., b.i.d.) did attenuate neutrophil infiltration. Data from CSE mice are represented by black bars, data from sham controls represented by white bars, data from the CSE with compound treatment in gray, diagonal-striped bars. Data plotted as the mean ± sem with an n = 7-10 for each group. Significance (* = p < 0.05, ** = p < 0.01, *** = p < 0.001) was determined versus smoke vehicle control group.

After 3 days of CSE, there was an increase in BALF neutrophil numbers in vehicle-treated mice compared to sham-exposed, vehicle-treated controls (p < 0.01) (figure 2A-C). Budesonide, administered i.n., had no effect on neutrophil numbers (Figure 2A). Conversely, roflumilast (Figure 2B) and fluvastatin (Figure 2C) administered p.o. significantly reduced the numbers of BALF neutrophils by 87 ± 5% and 71 ± 9%, respectively (p < 0.01).

### Effect of therapeutically administered anti-inflammatory compounds on CSE-induced chronic inflammation

**Figure 3:**
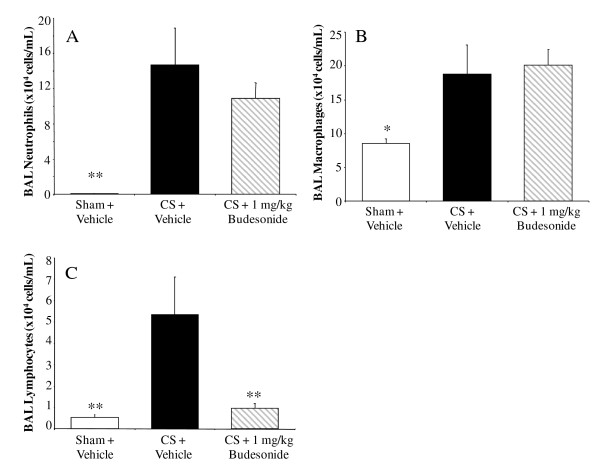
**The effect of budesonide on chronic CSE-induced inflammatory cell infiltrate**. After 14 weeks of CSE, budesonide (i.n., q.d.) had no effect on BALF neutrophil (A) and macrophage (B) numbers, whereas lymphocyte (C) numbers were reduced. Data from CSE mice are represented by black bars, data from sham controls represented by white bars, data from the CSE with compound treatment in gray, diagonal-striped bars. Data plotted as the mean ± sem with an n = 8-10 for each group. Significance (* = p < 0.05, ** = p < 0.01, *** = p < 0.001) was determined versus smoke vehicle control group.

**Figure 4:**
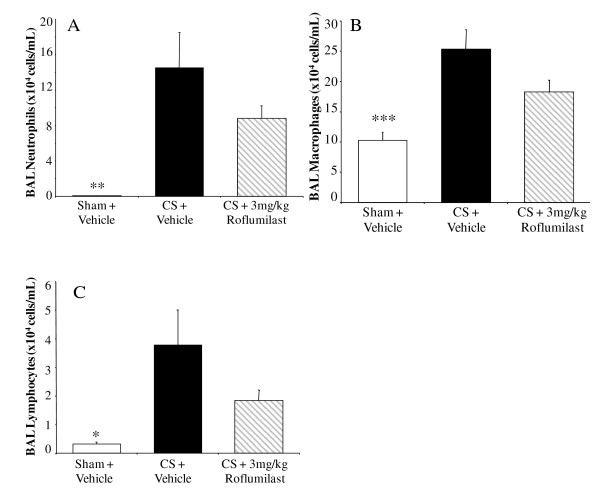
**The effect of roflumilast on chronic CSE-induced inflammatory cell infiltrate**. After 14 weeks of CSE, mice treated with roflumilast (p.o., q.d.) trended towards having reduced numbers of neutrophil (A), macrophage (B) and lymphocyte (C) in the BALF. Data from CSE mice are represented by black bars, data from sham controls represented by white bars, data from the CSE with compound treatment in gray, diagonal-striped bars. Data plotted as the mean ± sem with an n = 8-10 for each group. Significance (* = p < 0.05, ** = p < 0.01, *** = p < 0.001) was determined versus smoke vehicle control group.

**Figure 5:**
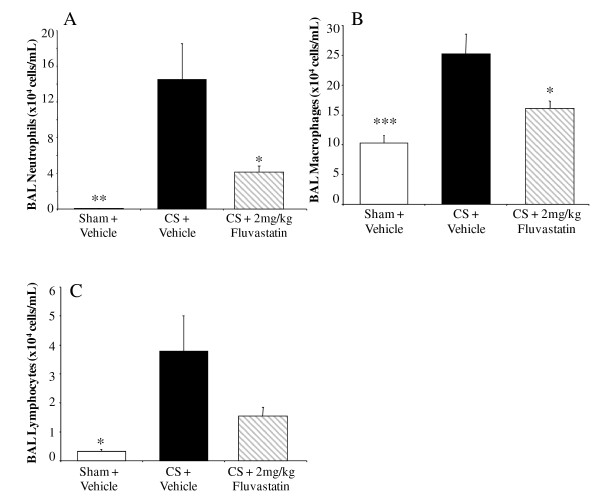
**The effect of fluvastatin on chronic CSE-induced inflammatory cell infiltrate**. Fluvastatin (p.o., b.i.d.) reduced CSE-induced neutrophil (A) and macrophage (B) infiltrate, but did not reduce the number of lymphocytes (C). Data from CSE mice are represented by black bars, data from sham controls represented by white bars, data from the CSE with compound treatment in gray, diagonal-striped bars. Data plotted as the mean ± sem with an n = 8-10 for each group. Significance (* = p < 0.05, ** = p < 0.01, *** = p < 0.001) was determined versus smoke vehicle control group.

Chronic CSE increased the numbers of BALF neutrophils, macrophages and lymphocytes in the all vehicle-treated groups compared to sham-exposed, vehicle-treated controls. Budesonide (1 mg kg^-1^, i.n., q.d.) had no effect on BALF neutrophil or macrophage numbers (Figure 3A and 3B). Budesonide did, however, reduce the number of lymphocytes recovered by 91 ± 4% (p < 0.01) (Figure 3C). Roflumilast trended towards reducing the increase in BALF neutrophils by 40 ± 10% (Figure 4A), macrophages by 47 ± 13% (Figure 4B) and lymphocytes by 56 ± 10% (Figure 4C); however these effects on BALF leukocyte numbers were not statistically significant. Fluvastatin reduced the number of neutrophils by 74 ± 5% (Figure 5A) and macrophages by 64 ± 7% (Figure 5B) in the BALF (p < 0.05), but the reduction of BALF lymphocytes was not statistically significant (Figure 5C).

## Discussion

These data confirm that there are different inflammatory phenotypes after either an acute or chronic CSE. The most obvious difference being the greater numbers and spectrum of inflammatory cell infiltrate present in the airways after a chronic exposure compared to the predominantly low-grade neutrophilic inflammation after an acute exposure. We also demonstrated that the acute (prophylactic) CSE models can be used to identify compounds with potential anti-inflammatory efficacy, but could not be used to predict the therapeutic efficacy of the same compounds on chronic CSE-induced inflammation. This is the first time the prophylactic and therapeutic effects of these 3 broad spectrum anti-inflammatory compounds have been assessed in these models. Again, we focused our assessment of efficacy around the numbers of inflammatory cells recovered in the BALF as this is a direct preclinical correlate to endpoints used in early proof-of-concept studies in man. Additionally, infiltrating inflammatory cells (particularly macrophages and lymphocytes) have been directly linked to the subsequent development of COPD-like lung pathologies in these modeling systems [18,19]. We did not assess levels of cytokines or chemokines in the BALF or lung tissue for several reasons. First, changes in the levels of these mediators are not acceptable biomarkers at the present time for studies conducted in COPD patients because they do not consistently track with disease progression. Second, we and others [20,21] have shown that the effects anti-inflammatory molecules (e.g. steroids) have on chemokine levels do not necessarily align with their ability to block cell infiltrates. Finally, investigating the molecular mechanisms responsible for the effects of these 3 compounds in the models was beyond the scope of these studies and (for the reasons just described) would require more than an assessment of cytokine or chemokine production. These data will, however, be important to collect in future studies elucidating the specific mechanisms of these compounds in these models.

The response to CSE in rodents has both an acute phase consisting of neutrophil infiltrate peaking after one week of exposures and a chronic phase consisting of neutrophils, macrophages and lymphocytes that begins after one month of exposures as previously reported by us and others [1,14]. Between weeks 1 and 4 the inflammation goes through a transition period, where neutrophil numbers decline, while macrophages and lymphocytes begin to increase, but not in a completely progressive fashion. After 1 month the inflammatory response is progressive, more pronounced, and eventually leads to airway remodeling and emphysema. We tested 3 mechanistically distinct anti-inflammatory compounds in both the 3-day and 14-week CSE models to determine whether these subtle differences in the inflammatory phenotype during each phase of the response affected compound efficacy.

In the acute models, CSE consistently induced an increase in the number of neutrophils recovered in the BALF and as such this remained the primary endpoint in the acute model. We and others have previously shown that glucocorticoids cannot affect the acute inflammatory changes induced by CSE at doses which can attenuate allergen-induced inflammation [2,9,13,22]. We confirmed our previous findings (conducted using BALB/C mice) here, using C57BL/6 mice as again budesonide had no effect on acute CSE-induced neutrophilia in this strain. Similarly, budesonide had no effect on chronic CSE-induced macrophage or neutrophil infiltration in the lung. There was, however, a profound effect on lymphocytic infiltrate that may be due to budesonide's effect on the thymus [23,24]; however, the mechanism for this effect on lymphocytes still requires further investigation. These findings reflect the inability of glucocorticoids to attenuate the inflammation observed in COPD patients. Additionally, the data suggest that the CSE models can be used for investigating mechanisms related to steroid-resistant inflammation and for identifying approaches that may be able to restore steroid efficacy in COPD [2].

Statins, on the other hand, have been reported to slow the rate of lung function decline and reduce mortality in COPD patients [25,26]; however, no one as yet has looked at whether statins affect the inflammation associated with the disease. Prophylactic administration of a statin (i.e., simvastatin) has previously been demonstrated to inhibit inflammation, emphysema and remodeling of the lung vasculature after chronic CSE in Sprague-Dawley rats [13]. It is unclear how statins act as anti-inflammatory agents, although their ability to block adhesion molecules and preventing the prenylation of proteins involved in inflammatory signaling (e.g. GTP-binding proteins) are well documented [27-29]. In our acute (prophylactic) system, fluvastatin attenuated acute neutrophilia induced by CSE. When we tested fluvastatin in the more chronic (therapeutic) model, it reduced the numbers of neutrophil and macrophage recovered in the BALF, while there only a modest reduction in lymphocyte infiltration, but the latter was not significant. These data are encouraging and imply that statins may prove to be effective anti-inflammatory treatments for COPD.

We also assessed the effect of a PDE4 inhibitor, roflumilast, in our models as it has previously been shown to reduce both acute and chronic CSE-induced inflammation in rodents when administered prophylactically at similar doses [11,12,16]. Here, we show that while roflumilast can reduce acute CSE-induced inflammation when given prophylactically, it failed to significantly reduce an established chronic inflammation when administered therapeutically. We propose that our results differ from those reported by Martorana and colleagues [11] due to the different dosing schedules (prophylactic versus therapeutic). Their results did, however, suggest that higher doses were needed to inhibit the chronic response. Our findings are in accordance with those reported by Le Quement and colleagues [16] who found that roflumilast reduced BALF neutrophils after 4 days of CSE, but could not attenuate the numbers of BALF macrophages after 11 days of CSE. The authors attributed these differences to PDE4 inhibitors' inability to inhibit macrophage activation and recruitment [16]. Our data from the chronic CSE system demonstrate that roflumilast does not effectively reduce inflammatory cell recruitment in general. These data, along with that reported by Le Quement and colleagues [16], do suggest that there are different mechanisms driving the acute and chronic phases of the inflammatory response. Roflumilast has demonstrated very limited efficacy in the clinic as well, which has largely been attributed to dose-limitation associated with roflumilast's side-effect profile. It has been reported that roflumilast can reduce the number of inflammatory cells recovered from COPD patients by approximately 30-50% [30]. This level of inhibition is consistent with what we observed in the chronic CSE experiment; however, these *in vivo *models are typically powered to identify a ≥ 50% inhibitory effect. As such, these observations suggest that the chronic model is a more rigorous assessment of a compound's anti-inflammatory efficacy that may be more reflective of the clinical situation.

## Conclusions

The data reported here demonstrate that overall, the prophylactic effects of compounds in the acute CSE models can identify compounds with anti-inflammatory efficacy; however, effects in acute, prophylactic systems did not reliably predict those observed in chronic models where compounds were administered therapeutically. This suggests that mechanisms that are involved in the initiation of CSE-induced inflammation may not be the same as those involved in the progression of the chronic response. Thus, we conclude that the acute CSE model is a robust, primary modeling system that can be used to assess the potential efficacy of candidate compounds, particularly those with broad spectrum anti-inflammatory effects or that target neutrophilic inflammation. However, testing candidate compounds in a chronic system more akin to the clinical situation where a progressive chronic inflammation (with a broader spectrum of inflammatory cell infiltrate) is already established in the lungs would always be prudent to get a more complete understanding of a compound's range of effects.

## List of abbreviations

COPD: Chronic obstructive pulmonary disease; CS: Cigarette smoke; CSE: Cigarette smoke exposure; BALF: Bronchoalveolar lavage fluid; p.o.:Per os (by mouth); i.n.: Intranasal; q.d.: Quaque die (once daily); b.i.d.: Bis in die (twice a day)

## Competing interests

The authors declare that they have no competing interests.

## Authors' contributions

W-YHW, AM, GK, WP, JM, DW, and CSS contributed to the acquisition and analysis of the data, have contributed to the drafting of the manuscript, read and approve of the final version of this manuscript. CSS designed the studies and drafted the manuscript.

## References

[B1] Stevenson CS Docx C Webster R Battram C Hynx D Giddings J Cooper PR Chakravarty P Rahman I Marwick JA Kirkham PA Charman C Richardson DL Nirmala NR Whittaker P Butler K Comprehensive gene expression profiling of rat lung reveals distinct acute and chronic responses to cigarette smoke inhalation Am J Physiol Lung Cell Mol Physiol 2007 293 L1183 L1193 (2007) 10.1152/ajplung.00105.2007 17720875

[B2] Marwick JA Caramori G Stevenson CC Casolari P Jazrawi E Barnes PJ Ito K Adcock IM Kirkham PA Papi A Inhibition of PI3K{delta} Restores Glucocorticoid Function in Smoking-induced Airway Inflammation in Mice Am J Respir Crit Care Med 2009 179 542 548 10.1164/rccm.200810-1570OC 19164702

[B3] Wright JL Churg A Cigarette smoke causes physiologic and morphologic changes of emphysema in the guinea pig Am Rev Respir Dis 1990 142 1422 1428 2252262 10.1164/ajrccm/142.6_Pt_1.1422

[B4] Vlahos R Bozinovski S Jones JE Powell J Gras J Lilja A Hansen MJ Gualano RC Irving L Anderson GP Differential protease, innate immunity and NFkB induction profiles during lung inflammation induced by sub-chronic cigarette smoke exposure in mice Am J Physiol Lung Cell Mol Physiol 2006 290 L931 L945 10.1152/ajplung.00201.2005 16361358

[B5] Churg A Dai J Tai H Xie C Wright JL Tumor necrosis factor-alpha is central to acute cigarette smoke-induced inflammation and connective tissue breakdown Am J Respir Crit Care Med 2002 166 849 854 10.1164/rccm.200202-097OC 12231496

[B6] Churg A Wang RD Tai H Wang X Xie C Wright JL Tumor necrosis factor-alpha drives 70% of cigarette smoke-induced emphysema in the mouse Am J Respir Crit Care Med 2004 170 492 498 10.1164/rccm.200404-511OC 15184206

[B7] Rennard SI Fogarty C Kelsen S Long W Ramsdell J Allison J Mahler D Saadeh C Siler T Snell P Korenblat P Smith W Kaye M Mandel M Andrews C Prabhu R Donohue JF Watt R Lo KH Schlenker-Herceg R Barnathan ES Murray J COPD Investigators The safety and efficacy of infliximab in moderate to severe chronic obstructive pulmonary disease Am J Respir Crit Care Med 2007 175 926 934 10.1164/rccm.200607-995OC 17290043

[B8] Stevenson CS Coote K Webster R Johnston H Atherton HC Nicholls A Giddings J Sugar R Jackson A Press NJ Brown Z Butler K Danahay H Characterization of cigarette smoke-induced inflammatory and mucus hypersecretory changes in rat lung and the role of CXCR2 ligands in mediating this effect Am J Physiol Lung Cell Mol Physiol 2005 288 L514 L522 10.1152/ajplung.00317.2004 15516486

[B9] Bonneau O Wyss D Ferretti S Blaydon C Stevenson CS Trifilieff A Effect of adenosine A2A receptor activation in murine models of respiratory disorders Am J Physiol Lung Cell Mol Physiol 2006 290 L1036 L1043 10.1152/ajplung.00422.2005 16339780

[B10] Morris A Kinnear G Wan WY Wyss D Bahra P Stevenson CS Comparison of cigarette smoke-induced acute inflammation in multiple strains of mice and the effect of a matrix metalloproteinase inhibitor on these responses J Pharmacol Exp Ther 2007 327 851 862 10.1124/jpet.108.140848 18806126

[B11] Martorana PA Beume R Lucattelli M Wollin L Lungarella G Roflumilast fully prevents emphysema in mice chronically exposed to cigarette smoke Am J Respir Crit Care Med 2005 172 848 853 10.1164/rccm.200411-1549OC 15961691

[B12] Leclerc O Lagente V Planquois JM Berthelier C Artola M Eichholtz T Bertrand CP Schmidlin F Involvement of MMP-12 and phosphodiesterase type 4 in cigarette smoke-induced inflammation in mice Eur Respir J 2006 27 1102 1109 10.1183/09031936.06.00076905 16510458

[B13] Lee JH Lee DS Kim EK Choe KH Oh YM Shim TS Kim SE Lee YS Lee SD Simvastatin inhibits cigarette smoking-induced emphysema and pulmonary hypertension in rat lungs Am J Respir Crit Care Med 2005 172 987 993 10.1164/rccm.200501-041OC 16002570

[B14] D'hulst AI Vermaelen KY Brusselle GG Joos GF Pauwels RA Time course of cigarette smoke-induced pulmonary inflammation in mice Eur Respir J 2005 26 204 213 10.1183/09031936.05.00095204 16055867

[B15] Maes T Bracke KR Vermaelen KY Demedts IK Joos GF Pauwels RA Brusselle GG Murine TLR4 is implicated in cigarette smoke-induced pulmonary inflammation Int Arch Allergy Immunol 2006 141 354 368 10.1159/000095462 16940747

[B16] LeQuement C Guenon I Gillon J-Y Valenca S Cayron-Elizondo V Lagente V Boichot E The selective MMP-12 inhibitor, AS111793 reduces airway inflammation in mice exposed to cigarette smoke Brit J Pharm 2008 154 1206 1215 10.1038/bjp.2008.180 PMC2483400 18493250

[B17] Ofulue AF Ko M Effects of depletion of neutrophils or macrophages on development of cigarette smoke-induced emphysema Am J Physiol 1999 277 L97 L105 10409235 10.1152/ajplung.1999.277.1.L97

[B18] Maeno T Houghton AM Quintero PA Grumelli S Owen CA Shapiro SD CD8+ T Cells are required for inflammation and destruction in cigarette smoke-induced emphysema in mice J Immunol 2007 178 8090 8096 17548647 10.4049/jimmunol.178.12.8090

[B19] Bandoh T Mitani H Niihashi M Kusumi Y Kimura M Ishikawa J Totsuka T Sakurai I Hayashi S Fluvastatin suppresses atherosclerotic progression, mediated through its inhibitory effect on endothelial dysfunction, lipid peroxidation, and macrophage deposition J Cardiovasc Pharmacol 2000 35 136 144 10.1097/00005344-200001000-00018 10630744

[B20] Stevenson CS Coote K Webster R Nicholls A Giddings J Butler K Danahay H An Acute Model of Cigarette Smoke-Induced Inflammation in Rat that is Partially Steroid-Insensitive Inflamm Res 2003 52 s85

[B21] Vernooy JH Bracke KR Drummen NE Pauwels NS Zabeau L van Suylen RJ Tavernier J Joos GF Wouters EF Brusselle GG Leptin modulates innate and adaptive immune cell recruitment after cigarette smoke exposure in mice J Immunol 2010 184 7169 7177 10.4049/jimmunol.0900963 20488786

[B22] Marwick JA Kirkham PA Stevenson CS Danahay H Giddings J Butler K Donaldson K Macnee W Rahman I Cigarette smoke alters chromatin remodeling and induces proinflammatory genes in rat lungs Am J Respir Cell Mol Biol 2004 31 633 642 10.1165/rcmb.2004-0006OC 15333327

[B23] Belvisi MG Wicks SL Battram CH Bottoms SE Redford JE Woodman P Brown TJ Webber SE Foster ML Therapeutic benefit of a dissociated glucocorticoid and the relevance of in vitro separation of transrepression from transactivation activity J Immunol 2001 166 1975 1982 11160246 10.4049/jimmunol.166.3.1975

[B24] Szelenyi I Hochhaus G Heer S Kusters S Marx D Poppe H Engel J Loteprednol etabonate: a soft steroid for the treatment of allergic diseases of the airways Drugs Today (Barc) 2000 36 313 320 12861354 10.1358/dot.2000.36.5.575043

[B25] Søyseth V Brekke PH Smith P Omland T Statin use is associated with reduced mortality in COPD Eur Respir J 2007 29 279 283 10.1183/09031936.00106406 17050558

[B26] Mancini GB Etminan M Zhang B Levesque LE FitzGerald JM Brophy JM Reduction of morbidity and mortality by statins, angiotensin-converting enzyme inhibitors, and angiotensin receptor blockers in patients with chronic obstructive pulmonary disease J Am Coll Cardiol 2006 47 2554 2560 10.1016/j.jacc.2006.04.039 16781387

[B27] Kimura M Kurose I Russell J Granger DN Effects of fluvastatin on leucocyte endothelial cell adhesion in hypercholesterolemic mice Arterioscler Thromb Vasc Biol 1997 17 e1521 e1526 10.1161/01.atv.17.8.1521 9301630

[B28] Bellosta S Via D Canavesi M Pfister P Fumagalli R Paoletti R Bernini F HMG-CoA reductase inhibitors reduce MMP-9 secretion by macrophages Arterioscler Thromb Vasc Biol 1998 8 1671 1678 10.1161/01.atv.18.11.1671 9812903

[B29] Wong B Lumma WC Smith AM Sisko JT Wright SD Cai TQ Statins suppress THP-1 cell migration and secretion of matrix metalloproteinase 9 by inhibiting geranylgeranylation J Leukoc Biol 2001 69 959 962 11404382

[B30] Grootendorst DC Gauw SA Verhoosel RM Sterk PJ Hospers JJ Bredenbröker D Bethke TD Hiemstra PS Rabe KF Reduction in sputum neutrophil and eosinophil numbers by the PDE4 inhibitor roflumilast in patients with COPD Thorax 2007 62 1081 1087 10.1136/thx.2006.075937 17573446 PMC2094292

